# A Novel Interprofessional Educational Experience for Internal Medicine Residents Within the Clinical Learning Environment

**DOI:** 10.7759/cureus.100207

**Published:** 2025-12-27

**Authors:** Jessica Donato, Bryce Montane, Chongliang Luo, Cecile Foshee

**Affiliations:** 1 Medicine, Cleveland Clinic, Cleveland, USA; 2 Internal Medicine, Washington University School of Medicine, St. Louis, USA; 3 Surgery, Washington University School of Medicine, St. Louis, USA

**Keywords:** curriculum implementation, experiential learning in medicine, graduate medical education (gme), internal medicine residency training, interprofessional education (ipe)

## Abstract

Introduction

The objective of this study was to evaluate the impact of a novel interprofessional education (IPE) curriculum on residents’ knowledge, skills, and attitudes related to interprofessional collaboration.

Methods

We implemented an innovative IPE prospective curriculum in our large academic internal medicine (IM) training program (2022-2023) for a cohort of first-year IM residents. The program aimed to enhance the knowledge of six healthcare professions (physical therapy, occupational therapy, speech/language pathology, respiratory therapy, registered nursing, and registered dieticians) while fostering interprofessional teamwork and communication. The curriculum was delivered over two days and included experiential learning and constructive small group debriefing sessions, whereby participants reflected on key learning points. A retrospective pre-post assessment instrument was implemented to assess self-reported knowledge, skill, and attitude changes using a five-point Likert scale. This design was chosen to reduce response shift bias. Success was defined as more than 50% of trainees showing improvement when comparing self-assessed scores before and after the educational experience. This proportion was chosen as it meant the majority of participants showed improvement in each metric. A total cohort of 101 residents participated in 2022 and 2023.

Results

Comparative pre-post analysis showed a statistically significant improvement in the knowledge of all six professions (with knowledge of occupational therapy showing the most improvement in approximately 98% of residents), effectiveness of working on an interprofessional team, effectiveness of communication, and overall effectiveness as physicians.

Conclusion

This initiative can be adapted in graduate medical education (GME) training programs that seek to improve and formalize IPE.

## Introduction

Interprofessional education (IPE) has been shown to enhance knowledge, skills, communication, and collaborative attitudes among healthcare providers, while also improving patient outcomes and satisfaction [1-5]. Generally, graduate medical education (GME) programs' IPE curricula emphasize nursing roles and responsibilities [1,3,6-9]. In a hospital setting, internal medicine (IM) trainees work with numerous healthcare professionals: physical and occupational therapists, speech and language pathologists, respiratory therapists, registered nurses, and registered dieticians [2,10-15]. Residency program directors across the United States have expressed an interest in expanding IPE in their programs, but time serves as a barrier for both teachers and learners [9,16]. We sought to develop a feasible, novel IPE program in our large academic IM training program to increase the knowledge of these healthcare professions and enhance interprofessional teamwork in the inpatient setting. This curricular report describes the development, implementation, and evaluation of a two-day IPE program for first-year IM residents, with the aim of improving trainees’ knowledge of six inpatient healthcare professions, as well as their self-reported interprofessional teamwork, communication, and perceived effectiveness as physicians.

## Materials and methods

**Figure 1:**
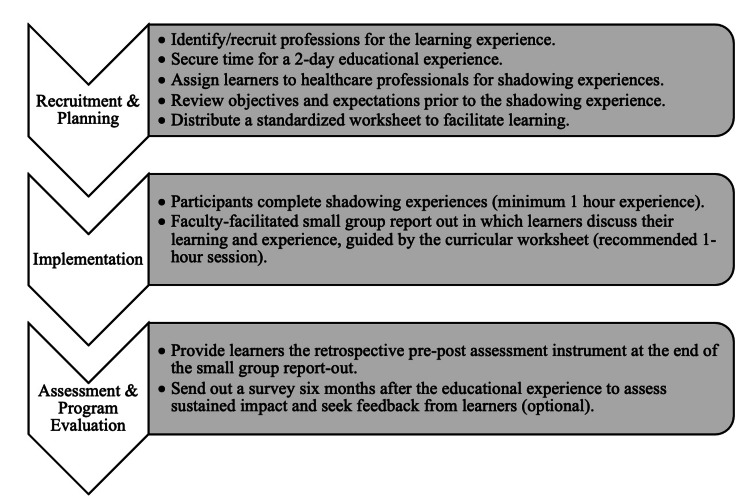
Overview of steps for curricular development and implementation

The planning period for the IPE program consisted of nine months, including recruitment of faculty from multiple professions: physical therapy (PT), occupational therapy (OT), speech and language pathology (SLP), respiratory therapy (RT), and registered nursing (RN). In 2023, registered dietician (RD) was added as a sixth profession. These professions were chosen as they frequently collaborate with residents in the inpatient setting. Our prospective IPE program was implemented from March 2022 to March 2023 for the IM training program at the Cleveland Clinic (Figure 1).

The curriculum was delivered over two days and incorporated experiential learning and peer teaching, consistent with principles of adult learning and social learning theory, within a small-group setting. The cohort included two separate resident classes (2022 and 2023) with nine to 12 residents per week, for five consecutive weeks.

Our IPE program sought to increase resident knowledge of six inpatient professions (PT, OT, SLP, RT, RN, RD) and enhance interprofessional teamwork/communication. This educational experience focused on first-year IM residents, six to nine months into their training.

On day one of the educational experience, we paired each resident with a faculty member from each profession (PT, OT, SLP, RT, RN, or RD). Each resident worked with a different provider, to ensure a unique experience, for approximately two hours. In this time, we asked the trainees to complete a one-page worksheet (Appendix A) about the observed profession that outlined the education, workload, workflow, responsibilities, indications for consultation, and communication preferences. The IM faculty met with leaders from each profession to review expectations, and worksheets for training purposes. To ensure consistency, approximately two faculty members from of each respective profession were allowed to work with trainees to limit the number of providers in this initiative for standardization purposes. On day two, all the residents discussed what they learned with their colleagues, with an IM faculty physician supervising the discussion using a facilitator guide (Appendix B). All the participants received a one-page summary table on all professions to consolidate learning points (Appendix C). Faculty to student ratios were 1:1 on day one and 1:4 on day two.

At the end of each report-out session, trainees were provided with a retrospective pre-post assessment instrument to reduce response shift bias (Appendix D) [17,18]. Consistent with published interdisciplinary initiatives, a five-point Likert scale was used (one being least and five being most), to assess self-reported changes in knowledge of the six professions, as well as skills and attitude related to interprofessional teamwork and communication [17,18]. The instrument also gathered comments about how the experience could change their own practice and roles on the inpatient medical team.

Our significance level, alpha value equal to 0.05, was adjusted for multiple comparisons, 11 in total, using false discovery rate correction (FDR) [19]. Data collected from the self-assessments was ordinal and a non-parametric outcome of interest was chosen: the mean proportion of residents who improved when comparing their respective pre- and post-assessment scores [17]. Success was defined as more than 50% of trainees (mean proportion of 0.5) that improved when comparing self-assessed scores before and after the curriculum. This proportion was chosen as it meant the majority of participants showed improvement in each metric. Our null hypothesis was the difference in post- and pre-assessment scores was less than or equal to zero, meaning the majority of residents (proportion of >0.5) would show no improvement. Proportions were calculated using the binomial theorem with 95% confidence intervals (CIs) for our primary outcome. Median, 25th percentile, and 75th quartile, and interquartile range (IQR) scores for each domain in the pre- and post-assessments were calculated for our ordinal data [20,21].

A follow-up survey was sent to the 2022 cohort six months after this educational experience to evaluate the impact of the educational experience on clinical practice and teamwork after transitioning to a senior resident role, using a Likert scale (strongly disagree, disagree, agree, strongly agree). This program was determined exempt from Institutional Review Board review (number: #24-707) given the use of deidentified instruments for educational and program evaluation purposes.

## Results

Forty-nine first-year residents participated in our 2022 pilot and 52 in the 2023 program, forming a total of 101 participants. Residents were paired with faculty from one of six professions: 10 to PT, 11 to OT, 13 to SLP, 20 to RT, 42 to RN, and five to RD. There was a 98% attendance (48/49 in 2022; 51/52 in 2023) at the in-person experience. The attendance at the report-out sessions was 94% (100% in 2022; 88% in 2023). The retrospective pre-post assessment instrument was completed by 93% (94/101) of trainees regarding self-reported knowledge (PT, OT, RT, RN), skills, and attitudes. The SLP knowledge pre-post-assessment was completed by 94% (95/101) of residents. A six-month follow-up survey was completed by 46% (24/52) of participants in the 2022 cohort. The RD knowledge assessment, available only for the 2023 participants, had an 88% (46/52) completion rate.

The outcome of interest was the proportion of residents who improved when comparing pre- and post-assessment Likert scores (Table 1).

**Table 1:** Proportion of improvement in interprofessional (IP) knowledge, skills, and attitudes Proportion of residents that improved on retrospective pre-post self-assessment test regarding their knowledge each respective profession. The same process was done to assess skills and attitudes (effectiveness of work on an IP team, effectiveness of communication, value of non-physician team members, value of IP team approach, and effectiveness in physician role because of knowledge of non-physician team members). PT: physical therapy, OT: occupational therapy, SLP: speech therapy, RT: respiratory therapy, RN: registered nursing, and RD: registered dietician; FDR: False Discovery Rate.

Knowledge/Skill/Attitude	Sample size	Mean proportion resident improvement	95% CI lower bound	95% CI upper bound	P value	Adjusted P value FDR
PT Knowledge	94	0.872	0.784	0.929	<0.001	<0.001
OT Knowledge	94	0.979	0.918	0.996	<0.001	<0.001
SLP Knowledge	95	0.957	0.888	0.986	<0.001	<0.001
RT Knowledge	94	0.883	0.796	0.937	<0.001	<0.001
RN Knowledge	94	0.809	0.712	0.88	<0.001	<0.001
RD Knowledge	46	0.891	0.756	0.959	<0.001	<0.001
Effective work on IP team	94	0.745	0.642	0.827	<0.001	<0.001
Effective communication	94	0.734	0.631	0.817	<0.001	<0.001
Value (non-physician members)	94	0.351	0.257	0.457	0.997	1
Value (IP team)	94	0.287	0.201	0.391	1	1
Effective physician	94	0.691	0.587	0.78	<0.001	<0.001

**Figure 2:**
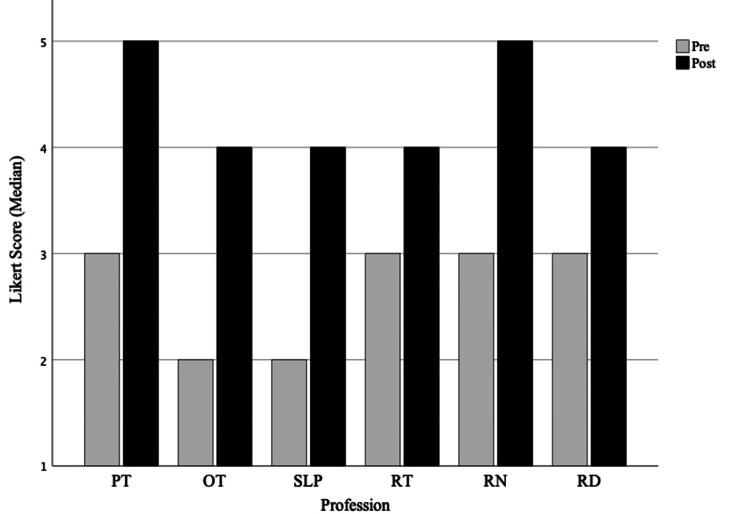
Change in the knowledge of professions A retrospective pre-post assessment instrument, using a 5-point Likert scale (1 being least and 5 being most), to assess self-reported changes in knowledge for the following professions: PT: physical therapy, OT: occupational therapy, SLP: speech therapy, RT: respiratory therapy, RN: registered nursing, and RD: registered dieticians. The median values for the pre-post assessment are labeled above.

A mean proportion above 0.5 means more than half of the participants improved in knowledge of the profession when comparing pre- versus post self-assessment scores. For PT, the mean proportion of residents who reported an increase in knowledge was 0.87 (95% CI 0.78 to 0.93, adjusted p-value <0.001). The mean proportion of residents who reported an improvement in OT knowledge was 0.98 (95% CI 0.92 to 1.00, adjusted p-value <0.001), 0.96 (95% CI 0.89 to 0.99, adjusted p-value <0.001) for SLP knowledge, 0.88 (95% CI 0.80 to 0.94, adjusted p-value <0.001) for RT knowledge, 0.81 (95% CI 0.71 to 0.88, adjusted p-value <0.001) for RN knowledge, and 0.89 (95% CI 0.76 to 0.96, adjusted p-value <0.001) for RD knowledge. Median Likert values for self-reported knowledge about the roles and responsibilities of all six professions are shown in Figure 2.

**Figure 3:**
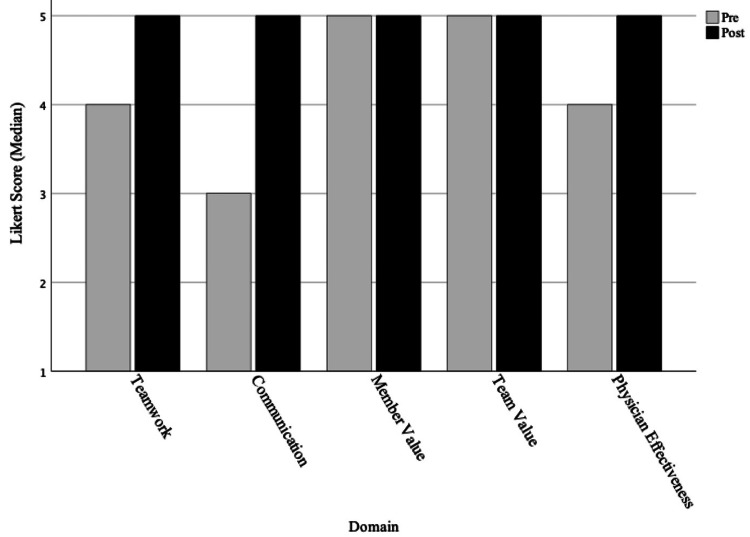
Changes in skills and attitudes A retrospective pre-post assessment instrument, using a 5-point Likert scale (1 being least and 5 being most), to assess self-reported changes in skills/attitudes regarding effectiveness of teamwork on an interprofessional (IP) team, effectiveness of communication, value of non-physician team members, value of IP team approach, and effectiveness in physician role because of knowledge of non-physician team members. The median values for the pre-post assessment are labeled above.

With regards to skills and attitudes (Table 1), the mean proportion of residents that improved was 0.75 (95% CI 0.64 to 0.83, adjusted p-value <0.001) for effectiveness of working on an interprofessional team, 0.73 (95% CI 0.63 to 0.82, adjusted p-value <0.001) for effective communication with an interprofessional team, 0.35 (95% CI 0.26 to 0.46, adjusted p-value 1.00) for valuing non-physician team members, 0.29 (95% CI 0.20 to 0.39, adjusted p-value 1.00) when assessing the value of an interprofessional team approach, and 0.69 (95% CI 0.59 to 0.78, adjusted p-value <0.001) for effectiveness as physicians. Median Likert values for self-reported skills and attitudes are shown in Figure 3.

Non-significant changes were seen for self reported values of both non-physician and interprofessional team approach to inpatient care.

**Figure 4:**
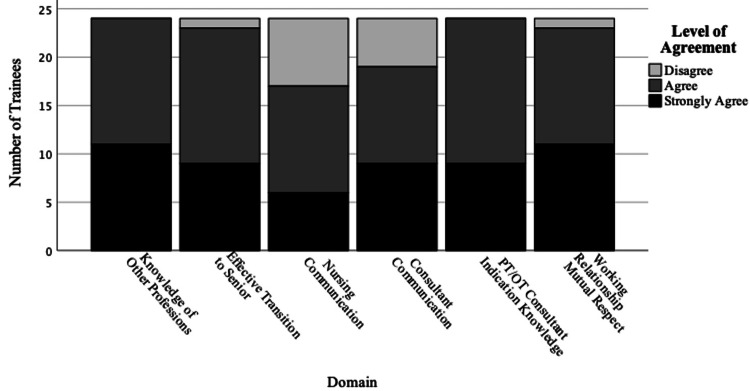
Skills and attitudes assessment (after six months) Results of six-month survey, using a 4-point Likert scale (strongly disagree, disagree, agree, strongly agree) showed sustained improvements in trainee-reported knowledge and skills from of this educational experience. None of the participants reported 'strongly disagree' for any domain.

From the 2022 cohort, a six-month follow-up survey showed that a vast majority reported sustained benefits from this educational experience. Most survey respondents agreed or strongly agreed that this educational experience increased their knowledge of other professions, made them more effective in transitioning to a senior resident role, improved communication with consultants and nurses, improved their understanding of appropriate indications for consultation, and contributed to better working relationships and mutual respect with other health professionals on the clinical team (Figure 4).

None of the participants in the six-month survey reported strong disagreement to any of the domains. 

## Discussion

This educational program describes an accessible way to incorporate IPE into GME programs. It expands beyond prior curricula that focused exclusively on physician-nursing collaboration by including other professionals like PT, OT, SLP, RT, and RD [1,3,6-8]. We saw a statistically significant increase in the proportion of residents who rated an increase in their knowledge, skills, and attitudes in almost every domain assessed. Two domains, valuing non-physician team members and an interprofessional team approach to inpatient care, showed no significant change, likely reflecting ceiling effects given high baseline ratings.

We believe this prospective curriculum was simple to implement, generalizable, and effectively built within the clinical learning environment. The experience allowed the trainees to immerse themselves in the care provided by their non-physician team members through direct observation and information gathering, with additional benefits of learning best practices to improve communication with each profession. By focusing on six different professions, our trainees had diverse experiences that led to robust opportunities for peer teaching and discussion during the report-out sessions. This allowed the trainees to get a more holistic view of their interprofessional teams, especially as it relates to less-familiar professions.

Limitations of this educational intervention include the use of self-reported changes in resident knowledge, skill, and attitude in a single IM residency program and may not be generalizable to smaller or community-based programs. This study is also limited to assessment of impact on residents only; we recommend assessing the impact on a diverse group of professionals in future studies. The study design and use of self-reported outcomes assessed in this study are prone to reporting, recall, and social desirability biases limiting the inferences that can be drawn from this data. Although False Discovery Rate (FDR) correction was applied to address multiple comparisons, analyses did not account for non-independence among repeated measures, which may contribute to residual Type I error inflation. Missing data was proportional across domain however it did impact the six-month follow-up survey results which limits long-term interpretation of findings. Lastly, this study was unable to assess patient-level or system-level outcomes.

This type of educational experience would be valuable across clinical contexts and training specialties, where interprofessional teamwork is universal [22]. In resource limited programs, this experience could be adapted to a single profession to improve feasibility. Formalizing IPE through a dedicated curriculum, incorporating it early in training, building the experience within the clinical learning environment, and having trainees learn directly from other professionals are foundational features for success [2,23]. This curricula could be expanded depending on the clinical context to include social workers, patient service representatives, and clinical pharmacists in an ambulatory setting [24,25].

## Conclusions

We developed an interprofessional curriculum for first-year IM residents at our institution and incorporated six healthcare professions (PT, OT, SLP, RT, RN, and RD). The goal was to foster the relationship between our trainees and other healthcare providers through experiential learning. Our novel IPE program shows trainees value interprofessional teamwork, and it is adaptable to enhance their knowledge and collaborative skills, based on the unique needs of a residency program. The fundamental components of our IPE experience would remain applicable in enhancing trainees’ ability to learn about their colleagues and work more effectively on interprofessional teams.

## Figures and Tables

**Figure 5:**
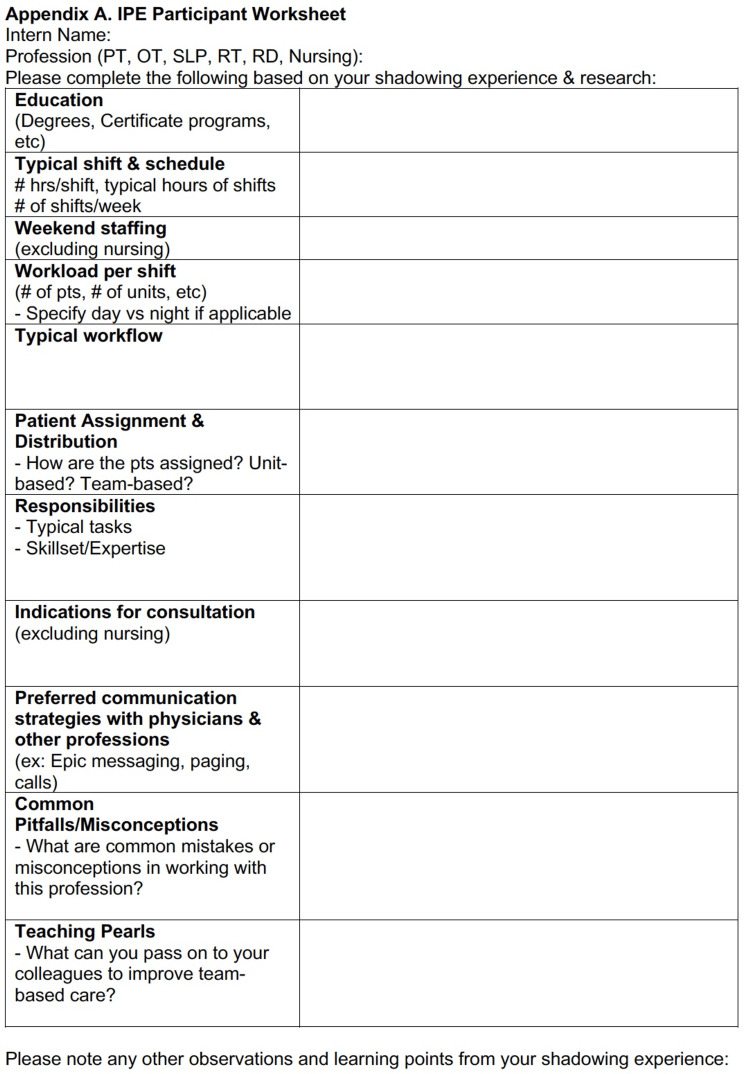
Interprofessional education participant worksheet

**Figure 6:**
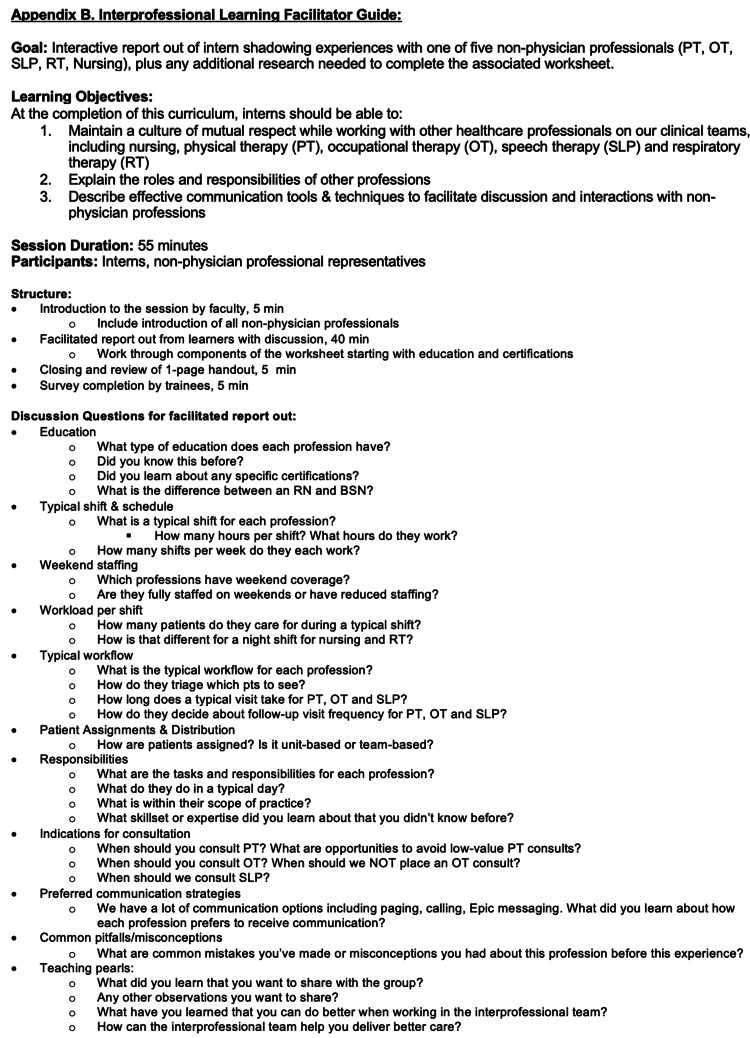
Interprofessional education facilitator guide day two

**Figure 7:**
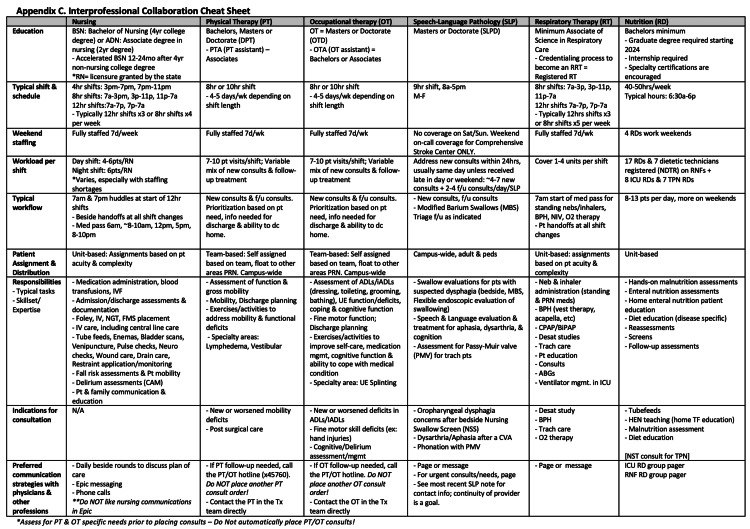
Interprofessional education summary table One-page summary table of consolidated learning points of each IPE profession.

**Figure 8:**
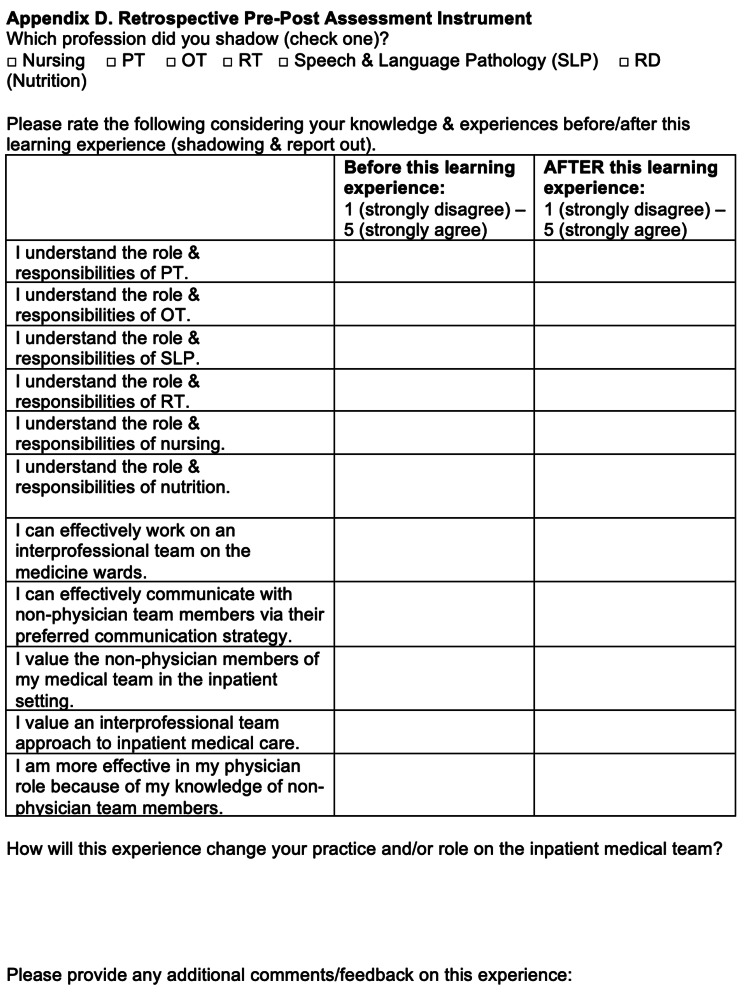
Retrospective pre-post assessment instrument

## Appendices

Appendix A 

Appendix B 

Appendix C 

Appendix D 
